# Prediction of gestational age based on genome-wide differentially methylated regions

**DOI:** 10.1186/s13059-016-1063-4

**Published:** 2016-10-07

**Authors:** J. Bohlin, S. E. Håberg, P. Magnus, S. E. Reese, H. K. Gjessing, M. C. Magnus, C. L. Parr, C. M. Page, S. J. London, W. Nystad

**Affiliations:** 1 Norwegian Institute of Public Health, P.O. Box 4404, 0456 Oslo, Norway; 2 National Institute of Environmental Health Sciences, National Institutes of Health, Department of Health and Human Services, PO Box 12233, MD A3-05, Research Triangle Park, Durham, NC 27709 USA

## Abstract

**Background:**

We explored the association between gestational age and cord blood DNA methylation at birth and whether DNA methylation could be effective in predicting gestational age due to limitations with the presently used methods. We used data from the Norwegian Mother and Child Birth Cohort study (MoBa) with Illumina HumanMethylation450 data measured for 1753 newborns in two batches: MoBa 1, n = 1068; and MoBa 2, n = 685. Gestational age was computed using both ultrasound and the last menstrual period. We evaluated associations between DNA methylation and gestational age and developed a statistical model for predicting gestational age using MoBa 1 for training and MoBa 2 for predictions. The prediction model was additionally used to compare ultrasound and last menstrual period-based gestational age predictions. Furthermore, both CpGs and associated genes detected in the training models were compared to those detected in a published prediction model for chronological age.

**Results:**

There were 5474 CpGs associated with ultrasound gestational age after adjustment for a set of covariates, including estimated cell type proportions, and Bonferroni-correction for multiple testing. Our model predicted ultrasound gestational age more accurately than it predicted last menstrual period gestational age.

**Conclusions:**

DNA methylation at birth appears to be a good predictor of gestational age. Ultrasound gestational age is more strongly associated with methylation than last menstrual period gestational age. The CpGs linked with our gestational age prediction model, and their associated genes, differed substantially from the corresponding CpGs and genes associated with a chronological age prediction model.

**Electronic supplementary material:**

The online version of this article (doi:10.1186/s13059-016-1063-4) contains supplementary material, which is available to authorized users.

## Background

Determination of gestational age (GA) is important for assessing due dates, giving adequate prenatal care, and suggesting appropriate interventions in preterm and post-term pregnancies. In Norway it is common clinical practice to assign pregnant women a due date based on the date of the last menstrual period (LMP). Around pregnancy week 18 a routine ultrasound examination, attended by almost all pregnant women, is used to calculate more precise estimates of GA and due date [1]. Although ultrasound-based estimates of GA are more precise in predicting the birth date than estimates based on the last menstrual period, LMP-based estimates may be preferred in some circumstances [2, 3]. There is wide variability in estimated GA even when ultrasound is used [4]. Ultrasound GA predictions eliminate uncertainties such as inaccurate reporting of the date of LMP and variability in the follicular phase length, although they assume uniform fetal growth during early pregnancy, which is only approximately true [5]. Hence, there is room for more accurate methods for estimating GA, which would be of great benefit in clinical practice.

There is growing interest in understanding the role of DNA methylation and its relation to GA in newborns. A study comprising 259 newborns, based on the Illumina HumanMethylation27 platform mapping approximately 28,000 CpG sites genome-wide and covering close to half the genes in the human genome, identified a number of candidate genes that were associated with GA [6]. Employing the CHARM 2.0 assay consisting of 2.1 million probes (covering 5.2 million probes arranged into probe groups) [7], Lee et al. performed a similar genome-wide screening, from a sample size of 187 newborns, and identified differentially methylated CpGs in the neighborhood of three genes (NFIX, RAPGEF2, and MSRB3) [8]. A more recent study, based on the Illumina HumanMethylation450 technology and longitudinal data from the Avon Longitudinal Study of Parents and Children (ALSPAC) cohort [9], found an association between DNA methylation and GA that appears to fade by early childhood [10]. Finally, birth weight correlates with GA and it has been shown that DNA methylation is associated with birth weight in newborns [11].

There has also been increased interest in exploring the relationship between DNA methylation and chronological age in humans. One study reported a fairly precise prediction of chronological age in humans using a small set of CpGs identified from several DNA methylation datasets [12]. These datasets were generated from many different studies, all of which were based on the Illumina HumanMethylation450 platform, assessing epigenetic effects from a multitude of cell types and disease-specific endpoints [12]. The strong relationship between DNA methylation and chronological age indicates that methylation analysis may be used as a tool in forensic age prediction [13]. Given the previous studies showing robust methylation signatures of GA, we reasoned that methylation data could also be used to predict GA.

The Norwegian Mother and Child Cohort Study (MoBa) [14, 15] contains genome-wide methylomes, as mapped by the Illumina HumanMethylation450 platform, for 1753 newborns. The 1753 newborns were sampled from the MoBa cohort and the methylation data were generated at two different points in time. The first batch, henceforth referred to as MoBa 1, containing 1068 newborns, was extracted and processed in 2011. The second batch, MoBa 2, which in the present study is only used for replication, includes 685 newborns and was processed in 2013. MoBa also had information on GA at birth estimated from both ultrasound measurements, near pregnancy week 18, as well as the LMP. Having a larger sample size than previous studies, we wanted to further explore the associations between GA and DNA methylation and the possibility of estimating GA, as has been demonstrated for chronological age [12]. We also examined whether the CpGs and genes that predict chronological age overlapped with CpGs and genes we found predictive for GA.

## Results and discussion

### Gestational age and methylation

**Fig. 1:**
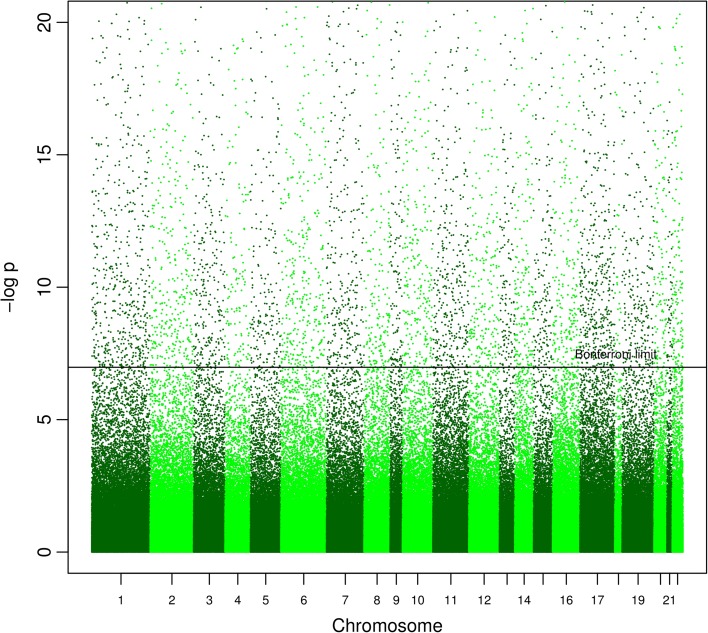
A Manhattan plot of regression model-based estimates of 473,731 CpGs (response) with ultrasound estimated gestational age as the explanatory variable. The regression model was adjusted for selection bias, offspring sex, maternal smoking, caesarian section, and estimated cell-type differences

**Fig. 2:**
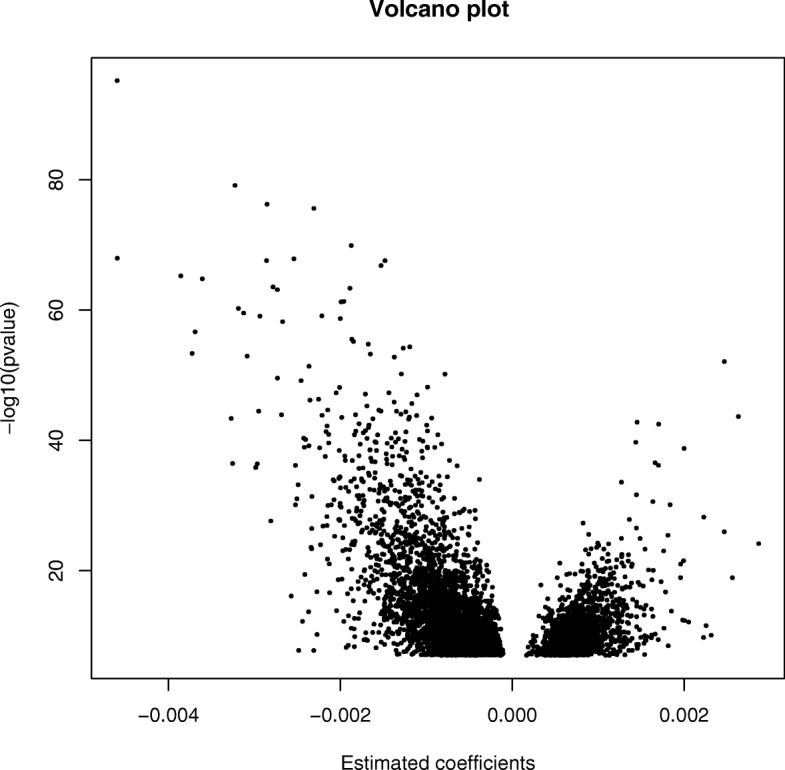
A volcano plot based on the 5474 significant CpG coefficient estimates (*horizontal axis*), resulting from the ultrasound estimated GA-based regression models, plotted against the corresponding *p*_*B*_ values (*vertical axis*). Negative coefficient estimates indicate decreased methylation, while positive estimates designate increased methylation

Robust MM-type linear regression was performed on MoBa 1 with CpG sites (β values) as the outcome variables. After correcting for a set of covariates (see the “Methods” section and Table 1; Table 2 contains the corresponding covariate statistics for the MoBa 2 dataset), we found approximately 5474 differentially methylated CpGs associated with GA calculated using ultrasound (10,784 CpGs when LMP-based estimations were used) after adjusting for multiple testing using Bonferroni correction (*p*_*B*_ < 0.05, 473,731 tests; Fig. 1). Figure 2 indicates that the significant CpGs were predominantly decreased in methylation (3911 versus 1563), which may suggest that increasing GA is associated with increased expression of genes linked to the differentially methylated probes as the CpGs mapped by the Illumina HumanMethylation450 platform are predominantly located in the promotor regions [16]. Using the less strict false discovery rate (FDR) correction for multiple testing [17], 44,359 probes (44,544 probes for LMP estimations) were found to be significant (q < 0.05, 473,731 tests). All statistical analyses described here were performed using the MoBa 1 dataset. More information regarding these CpGs can be found in Additional file 1: Tables S1 and S2.

**Table 1:** Covariates used in the preliminary regression models—MoBa 1

Covariate	Occurrence/mean value	N
Child’s sex, male	53.2 %	568/1068
Mean age of mother at birth	29.9 (95 % CI 29.7–30.2)	1068
Maternal smoking during pregnancy	14.6 %	156/1068
Caesarian section	11.5 %	123/1068
Asthma at 3 years	32.9 %	351/1068
Ultrasound estimated GA	279.6 (95 % CI 279–280.3)	1048
LMP estimated GA	282.3 (95 % CI 281.6–283.00)	1030

*CI* confidence interval

**Table 2:** Study population —MoBa 2

Covariate	Occurrence/mean value	N
Child’s sex, male	56.1 %	384/685
Mean age of mother at birth	30.0 (95 % CI 29.7–30.3)	685
Maternal smoking during pregnancy	10.2 %	70/685
Caesarian section	13 %	89/685
Asthma at 3 years	21.3 %	104/489
Ultrasound estimated GA	279.4 (95 % CI 278.5–280.2)	644
LMP estimated GA	281.5 (95 % CI 280.7–282.4)	615

*CI* confidence interval

### GA prediction by ultrasound and LMP estimates

**Fig. 3:**
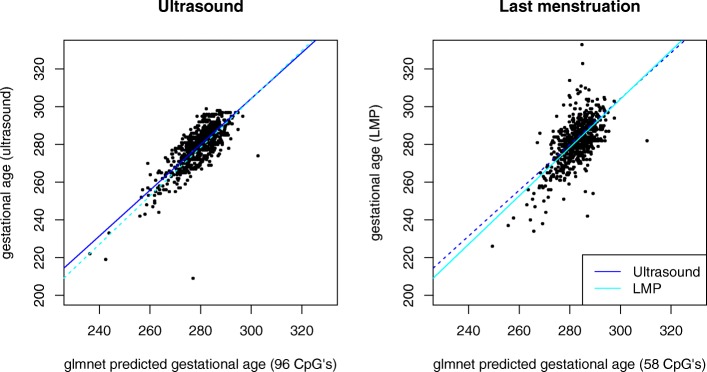
Output from the regression models with ultrasound- and LMP-estimated gestational age (GA) as the response (*vertical axis*) and predicted GA as the explanatory variable (*horizontal axis*) in the left and right panels, respectively. The models, which were based on 96 (ultrasound) and 58 (LMP) CpGs, were trained using 1068 samples from MoBa 1. Prediction was carried out on 685 samples from MoBa 2. The *dotted lines* represent the adjacent regression estimates

To facilitate prediction of GA, we used Lasso regression from the elastic net package “glmnet” [18] (see “Methods” for more details on how and which regression models were tested). The Lasso-based regression model was trained with several different constellations of the MoBa 1 dataset. To reduce biases from factors assumed to influence GA prediction, we first trained the regression model with the FDR and Bonferroni-significant CpGs from both LMP- and ultrasound-based regression models discussed in the previous section. In addition, we trained Lasso-regression models with the complete MoBa 1 dataset comprising 1068 samples (newborns) and 473,731 CpGs. The complete MoBa 2 dataset, containing 685 samples and 473,731 CpGs, was subsequently used for prediction of GA with the trained Lasso regression models. The Lasso-based GA predictions for both LMP and ultrasound were respectively compared to the LMP- and ultrasound-based GA estimations obtained from the MoBa cohort study using MM-type robust regression. As can be seen from Table 3, the performance of the LMP-based models was notably inferior to that of the ultrasound-based models in terms of both model fit (R^2^) and standard error measured as days within a 95 % prediction interval. All Lasso models were comparable in performance but those trained with the complete MoBa 1 dataset retained substantially fewer CpGs than the other models; therefore, our primary focus will be on these models. A visualization of the performance of the MoBa 1 trained model can be seen in Fig. 3. Information regarding the CpGs used in the respective prediction models can be found in Table 4 and Additional file 1: Table S3.

**Table 3:** GA prediction results

	R^2^	SE	Number of CpGs
Ultrasound (Bonferroni adjusted)	0.65	12.7	105
LMP (Bonferroni adjusted)	0.52	14.6	84
Ultrasound (FDR adjusted)	0.67	12.4	132
LMP (FDR adjusted)	0.53	14.5	107
Ultrasound (MoBa 1)	0.66	12.5	96
LMP (MoBa 1)	0.5	14.9	58

Boferroni/FDR refers to Lasso models trained with CpGs from the regression model adjusted for a set of covariates, including cell-type, as well as multiple testing. MoBa 1 models were trained using the complete MoBa 1 methylation data. The R^2^ column shows the goodness-of-fit statistics, based on MM-type robust regression, followed by standard error (SE) in ± days (95 % prediction interval) and the rightmost column designates the number of CpGs retained in each model

**Table 4:** Number of CpGs associated with the different prediction/regression models

Ultrasound CpGs *p*_*B*_ < 0.05 (FDR q< 0.05)	5474 (44,359)
LMP CpGs *p*_*B*_ < 0.05 (FDR q< 0.05)	10,784 (44,544)
Common (intersect)	3654
Total unique CpGs (union)	12,604
Ultrasound predictor CpGs	96
LMP predictor CpGs	58
Overlapping predictor CpGs	23
Total unique predictor CpGs	131
Ultrasound unique predictor CpGs	73
LMP unique predictor CpGs	35

### Previously identified GA-related CpGs and genes

As mentioned, previous studies have identified a number of significant CpGs with respect to GA. These studies were carried out using different DNA methylation assays, such as the previous generation Illumina HumanMethylation27 platform [6], the CHARM 2.0 assay [7], and more recently the Illumina HumanMethylation450 platform [10]. The number of individuals used in these studies was considerably smaller than in the present, with the exception of the Aries (ALSPAC)-based study, which included up to 974 samples [10]. Nevertheless, a total of 260 CpGs (representing 183 unique genes) were reported from both the Schroeder et al. [6] and Aries [10] studies, which was considerably fewer than the number of CpGs identified in the present study (Table 4). Out of the total 3654 CpGs found to be differentially methylated with respect to GA in the MoBa cohort, 223 CpGs (linked to 138 genes) out 260 CpGs were found to overlap with those found by the previous two studies (130 genes overlapped with the 183 genes from the Schroeder et al. and Aries studies). However, out of the 223 overlapping CpGs found to be significantly differentially methylated with respect to GA in our study, only 26 CpGs (associated with 17 unique genes) were found to overlap with the CpGs used in the ultrasound/LMP-based prediction models, with only ten CpGs (covering five genes) overlapping among these again (Additional file 1: Table S4). It should be noted that the Schroeder et al. study [6] was based on the Illumina HumanMethylation27 platform and one CpG (cg20337106 mapping to gene C6orf139) was not present amongst the CpGs mapped by the HumanMethylation450 platform. See Tables 4 and 5 (as well as Additional file 1: Tables S4) for more details.

**Table 5:** Overlapping CpGs with previous studies

Total unique ultrasound and LMP CpGs *p*_*B*_ <0.05	12,604
Significant CpGs in Schroeder/Aries studies	260
Overlapping ultrasound and LMP CpGs with Schroeder and Aries studies	223
Unique predictor CpGs from both ultrasound/LMP models	131
Overlapping predictor CpGs with Schroeder and Aries	26

The number of CpGs associated with GA in our study found to overlap with CpGs in other studies on GA

The Lee et al. study [8] reported three genes, NFIX, MSRB3, and RAPGEF2, associated with GA. Since that study used the Charm 2.0 platform [7], which is based on a different technology to the Illumina HumanMethylation450 BeadChip employed in the present study, we checked for the presence of CpG sites, mapped by the Illumina platform, linked to the respective genes reported. Twelve CpGs were found for the NFIX gene, four for MSRB3 gene, and one for the RAPGEF2 gene amongst the total of 12,604 unique Bonferroni significant CpGs for both ultrasound- and LMP-based regression models. Out of the 131 unique significant CpGs employed in both ultrasound- and LMP-based GA prediction models, no overlapping genes were found with the genes identified by the Lee et al. study. Hence, while the genes reported by Lee et al. were found to have differentially methylated CpGs in our study, CpGs associated with these genes were not used in either the ultrasound- or LMP-based GA prediction models.

The 96 CpGs used to train the ultrasound-based GA prediction model mapped to 64 unique genes, while the 58 CpGs used to train the LMP-based GA prediction model mapped to 43 unique genes. Twenty-two genes were found to overlap between the ultrasound- and LMP-based prediction models. See Additional file 1: Tables S5 for more details regarding overlapping genes. The low number of overlapping CpGs between our ultrasound and LMP GA prediction models as well as the above-mentioned studies is puzzling and indicates that the association between GA and DNA methylation may be, more generally, linked to genome-wide development and/or changes to white blood cell profiles, which vary in ratio between cord blood and peripheral blood [10]. Cord blood is also known to contain stem cells and an increased number of stem cells that change DNA methylation profiles during gestation would most likely correlate with GA [19]. We did, however, try to correct for putative cell type influences using both principal components [20] and the method described by Houseman [21] but with negligible effect with respect to the performance of the prediction models.

### Gene ontology

The 22 genes found to overlap for both the ultrasound and LMP prediction models were examined with both DAVID (updated May 2016) [22, 23] and GOrilla (updated June 25, 2016) [24] databases in order to shed more light on the putative epigenetic influences responsible for the fairly accurate GA predictions observed in our study. However, FDR-significant associations were not identified with either DAVID or GOrilla (q > 0.35) and since the results from DAVID did not add anything to those from GOrilla, we only present results from the latter. GOrilla identified “Nuclear matrix” (GO:0016363, genes “NCOR2”, “DNMT3A”, “HLCS”, *p* = 2.32E-4), “Chromatin” (GO:0000785, genes “NCOR2”, “HGMA1”, “DNMT3A”, “HLCS”, *p* = 6.19E-4) (in the component ontology) “Positive regulation of cellular senescence” (GO:2000774, genes “HMGA1”, “YPEL3”, *p* = 2.49E-5) “Positive regulation of cell aging” (GO:0090343, genes “HMGA1”, “YPEL3”, *p* = 3.48E-5), “Cellular response to ethanol” (GO:0071361, genes “DNMT3A”, “ADCY7”, *p* = 9.08E-5), “Negative regulation of gene silencing” (GO:0060969, genes “NCOR2”, “HMGA1”, *p* = 2.51E-4), “Regulation of cellular senescence” (GO:2000772, genes “HMGA1”, “YPEL3”, *p* = 4.9E-4) and “Regulation of cell aging” (GO:0090342, genes “HMGA1”, “YPEL3”, *p* = 8.05E-4)“ (in the process ontology). While not FDR-significant, it was of interest to note that several of the pathways identified were related to cell aging and cellular senescence.

### Gestational age and chronological age

The prediction model for chronological age previously mentioned [12] was also based on a glmnet-trained model using 353 CpGs from the same Illumina HumanMethylation450 platform employed in the present study. These CpGs were compared to the ones used in both our ultrasound- and LMP-based GA prediction models. Surprisingly, we found that only one CpG (cg08965235 associated with the gene LTBP3) common to both ultrasound GA and chronological age prediction models (only cg08965235 and cg11299964, respectively, associated with the genes LTBP3 and MAPKAP1 for the LMP GA prediction model). Since multiple CpGs mapped by the Illumina HumanMethylation450 BeadChip platform are linked to each gene, we also examined the number of overlapping genes and found one for the ultrasound-based prediction model, LTBP3 (two for LMP-based prediction model: LTBP3 and TOM1L1). The difference between GA- and chronological age-related DNA methylation has been examined in greater detail in the Aries cohort study, which included DNA methylation data from cord blood as well as for ages 7, 15, and 17 years [10]. Furthermore, the substantially larger number of unique genes linked to the CpGs used by the respective prediction models (297, 64, and 43 genes for the chronological age and ultrasound and LMP GA prediction models, respectively) may suggest that different mechanisms linked to aging may be operating throughout life, something also mentioned in the Aries study [10]. The CpGs and genes that differed between the GA and chronological age prediction models can also be seen in Additional file 1: Tables S5.

### Practical implications

The prediction model described here is based on DNA extracted from cord blood from newborns. Our GA predictor establishes a more assured link between GA and methylation, which may have important applications. For instance, since GA is related to many childhood health outcomes, being able to assess GA in circumstances where it is uncertain or unresolved could be critical for effective treatment. Furthermore, blood spots from newborns are routinely stored and can be useful to study *in utero* factors related to childhood disease. For such studies, GA can be determined by using methylation analyses and, thus, be adjusted for when a potential confounder. Although we used a class of models that are widely referred to as “prediction” models, for this purpose, we are “estimating” GA after birth rather than predicting it in advance. For true prediction before birth, one would need fetal blood while still *in utero*, which is difficult to obtain and extraction of which is associated with high risk. Using fetal cells in the maternal circulation, however, the method presented here might eventually enable actual prediction of GA and the due date in pregnant women.

## Conclusions

We found that DNA methylation signatures at birth were strong predictors of GA. In addition, the prediction was more precise for ultrasound GA than for LMP GA. This is not unexpected, however, since ultrasound is generally regarded as a more reliable way to estimate GA amongst practitioners. Genes associated with chronological age do not appear to be strongly linked to genes associated with GA, suggesting that different epigenetic mechanisms are at work during different stages of life.

## Methods

### Study population

#### MoBa cohort

For this analysis, we included 1068 + 685 newborns sampled at different occasions from the Norwegian Mother and Child Cohort Study (MoBa), which has previously been described in detail [11, 14, 15] (Tables 1 and 2). The data collection in MoBa is approved by the Norwegian Data Inspectorate and the Institutional Review Board of the National Institute of Environmental Health Sciences, National Institute of Health, USA. The current study was also approved by the Regional Committee for Medical and Health Research Ethics of South East Norway.

### Variables

GA estimates at birth were collected from the Medical Birth Registry of Norway, for which it is mandatory for health professionals at birth clinics to report birth outcomes. The birth registry provides two estimates for pregnancy length (GA), one based on ultrasound measurements from around week 18 of pregnancy and one based on the maternally reported LMP. The birth registry also provided data on maternal age. Information on maternal education was obtained from the MoBa questionnaire for early pregnancies completed by the mother or based on data from the Medical Birth Registry of Norway (MBRN) [14, 15]. Additional details regarding the covariates particular to MoBa 1 in the present study can be found in Table 1 (Table 2 contains similar information for the MoBa 2 covariates).

### Pre-processing and quality control of methylation datasets

#### MoBa 1

The methylation data set used for training the prediction models consisted of 1204 Illumina HumanMethylation450 arrays based on cord blood DNA, each having 485,577 probes before quality control. Only singletons from unique mothers were included. Arrays not fulfilling the 5 % detection *p* value were removed together with all duplicates. Examination of plate effects revealed no bias; therefore, between-array normalization was not performed. However, within-array normalization was carried out using BMIQ from the wateRmelon package [25] to calibrate bias in type I and II probe technology. In addition to the Illumina control probes, we removed all probes on the X and Y chromosomes, resulting in total of 1068 arrays each consisting of 473,731 probes. For complete information and details regarding quality control of the Illumina HumanMethylation450 data used in the present study see [11, 26].

#### MoBa 2

This replication dataset consisted of the methylomes from 864 newborns each of which contained 485,577 probes. Only singletons from unique mothers were included in the dataset. All duplicates were removed and arrays not fulfilling the 5 % detection *p* value were excluded. Examination of the dataset revealed some mild-to-moderate plate effects; therefore, ComBat, utilizing empirical Bayes methodology, from the R package sva [27], was used for between-array normalization. Within-array normalization of type I and II probes was performed using BMIQ from the R package wateRmelon [25]. After exclusion of probes associated with the X and Y chromosomes, the Illumina control probes, and probes not found in the MoBa 1 dataset, the total number of probes was 473,731 for each of the 685 samples retained. Complete information regarding preparation of cord blood and quality control can be found in recent publications by Joubert et al. [28, 26].

### Statistical analysis

MM-type robust linear regression [29] was first performed on the MoBa 1 newborn methylomes, with β values (0 ≤ β ≤ 1) from each of the 473,731 CpG sites as outcome variables. GA reported for the MoBa cohort (ultrasound/LMP) was the explanatory variable. The regression models were adjusted for a set of covariates believed to be potential confounders. These included cell type composition estimates based on the Houseman procedure [21] (as computed from the minfi package with the Reinius dataset used as reference [30, 31]), child’s sex, maternal smoking, maternal age, study design (asthma diagnosed later at three years yes/no) and caesarian section (yes/no). Gestational age was based on (a) ultrasound measurements at around pregnancy week 18 and (b) reported LMP. Details regarding these covariates can be found in Tables 1 and 2 for both MoBa 1 and MoBa 2 datasets. We found 5474 CpGs to be significant (*p*_*B*_ < 0.05) for ultrasound-estimated GA (10,784 CpGs for LMP-estimated GA).

### Prediction models

The 5474 and 10,784 CpG probes found to be significant (*p*_*B*_ < 0.05; as well as 44,359 and 44,544 FDR-significant (q < 0.05) CpG probes) for ultrasound- and LMP-estimated GA from the regression models discussed in the previous section were added as predictors to “glmnet” elastic net models with α set to 0, 0.5, and 1. The most appropriate α (i.e., type of regression method) was found using leave-one-out cross-validation. Lasso-type regression (α = 0) resulted consistently in lower mean-squared error and was therefore our method of choice for the GA prediction models. The models based on α values of 0.5 and 1 where therefore not pursued further. Estimated Lasso penalties (also found using leave-one-out cross-validation) of 1 standard error above the minimum (λ_1se_), as suggested by Breiman [32], were preferred to minimum Lasso-penalties (λ_min_) as the latter penalties resulted in models with only a marginally higher coefficient of determination (R^2^) than the former at the cost of retaining a substantially larger set of CpGs. To test the model’s predictive abilities, we trained the Lasso model on all 1068 MoBa 1 samples with Bonferroni- and FDR-significant CpGs obtained from the regression model discussed in the previous section. In addition, the Lasso model was trained with the complete MoBa 1 dataset containing 473,731 CpGs and 1068 newborns. The corresponding CpGs from the 685 newborns in the MoBa 2 dataset were subsequently added to the trained Lasso models, which then returned estimated GAs for each newborn. All Lasso models were run with the same seed (1999). The predicted GAs for the 685 newborns from the Lasso model were regressed against the GA estimations reported by the MoBa cohort (response variable) using MM-type robust regression, the result of which can be observed in Fig. 3. The prediction model accuracy, in days, was reported as the 95 % prediction interval based on the robust regression models (Table 3).

Since the “glmnet” model will not run with missing values, all missing GA estimations and probes were imputed using impute from the Hmisc R-package (median imputation) [33]. The CpGs used in the prediction model for chronological age are freely available as supplementary material from the study by Steve Horvath [12]. Further information regarding the CpGs used in the GA prediction models can be found in Additional file 1: Tables S3.

## Additional file

Additional file 1: An Excel file containing information regarding CpGs and genes discussed in the present study. **Table S1.** All *p*_*B*_ < 0.05 and q < 0.05 significant CpGs associated with ultrasound-estimated gestational age. **Table S2.** All *p*_*B*_ < 0.05 and q < 0.05 significant CpGs associated with last menstrual period-estimated gestational age. **Table S3.** All unique CpGs used in both ultrasound and LMP prediction models. **Table S4.** CpGs reported by the Aries and Schroeder et al. studies overlapping with the CpGs used in the prediction models of the present study. **Table S5.** Information regarding genes associated with the CpGs from the different prediction models. (XLSX 8210 kb)
